# Kitchen elbow sign predicts surgical outcomes in adults with spinal deformity: a retrospective cohort study

**DOI:** 10.1038/s41598-021-92520-5

**Published:** 2021-06-18

**Authors:** Shizumasa Murata, Hiroshi Hashizume, Keiji Nagata, Yasutsugu Yukawa, Akihito Minamide, Hiroshi Iwasaki, Shunji Tsutsui, Masanari Takami, Ryo Taiji, Takuhei Kozaki, Hiroshi Yamada

**Affiliations:** 1 Department of Orthopedic Surgery, Wakayama Medical University, 811-1 Kimiidera, Wakayama City, Wakayama 641-8510 Japan; 2 Spine Center, Dokkyo Medical University Nikko Medical Center, 632 Takatoku, Nikko City, Tochigi 321-2593 Japan

**Keywords:** Medical research, Signs and symptoms

## Abstract

Kitchen elbow sign (KE-Sign) is a skin abnormality on the extensor side of the elbow and forearm that is often observed in patients with adult spinal deformity (ASD). The significance of KE-Sign in surgical cases was investigated. Overall, 114 patients with ASD treated with long spinal fusion were reviewed and divided into KE-Sign positive and negative groups. The preoperative and 1-year follow-up evaluations included radiographic parameters [C7 sagittal vertical axis (SVA), pelvic incidence (PI) and lumbar lordosis (LL)], the Oswestry Disability Index (ODI), visual analogue scales (VASs) for low back pain, leg pain, and satisfaction, and Short Form 36 questionnaire (SF-36). Multi-regression analysis was performed to identify patient satisfaction predictors and improvement in the ODI as dependent variables and preoperative background factors as independent variables. Preoperative characteristics showed no significant difference between both groups. Improvement in the ODI and VAS for satisfaction were significantly superior in the KE-Sign positive group. In multiple regression analysis, KE-Sign and preoperative ODI were significantly associated with improvement in the ODI; age, KE-Sign, preoperative low back pain VAS, and leg pain VAS were significantly associated with satisfaction. KE-Sign can be a predictor of better surgical outcomes in ASD patients.

## Introduction

**Figure 1:**
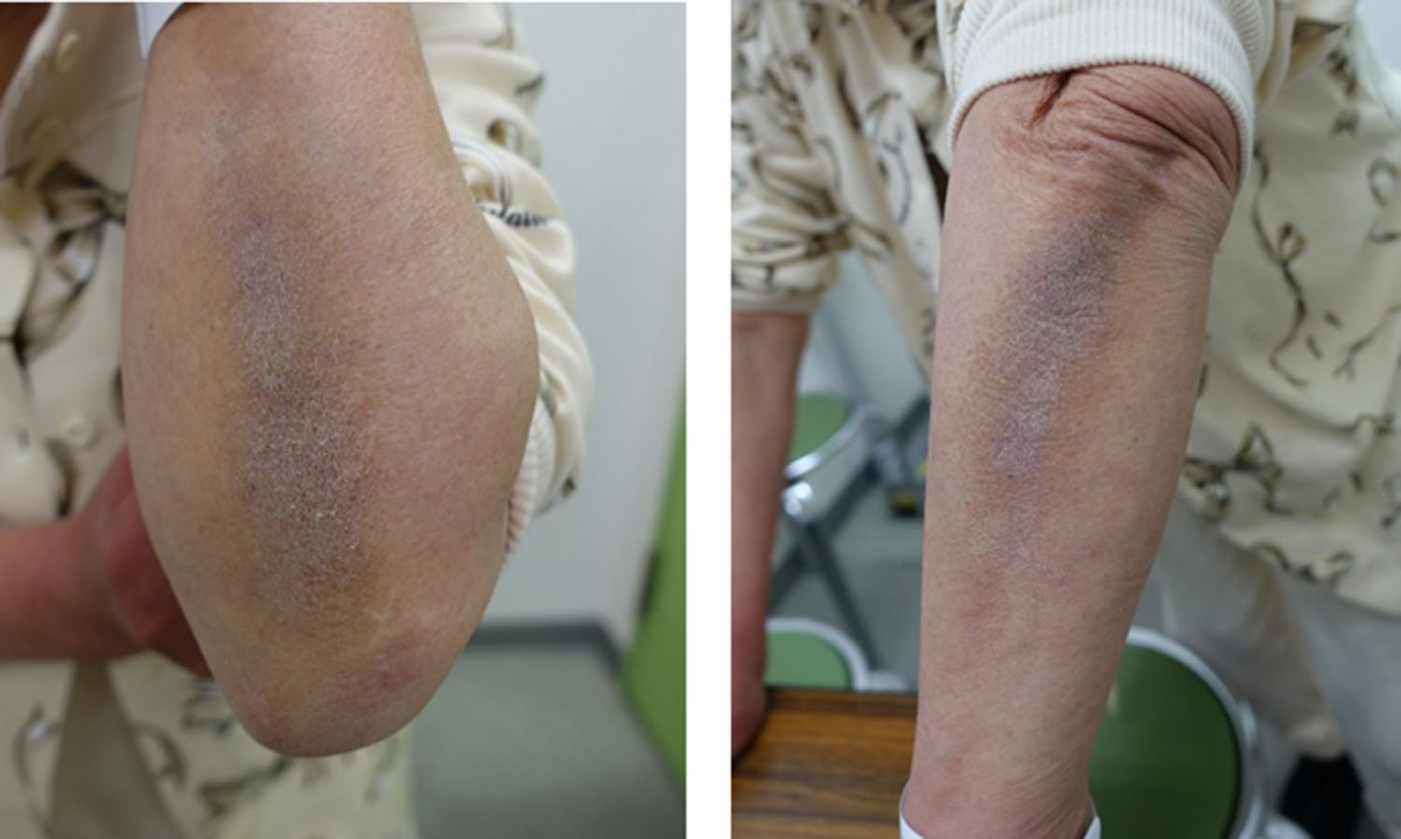
Kitchen elbow sign (KE-Sign).

In most developed countries, the average human life expectancy has doubled over the past 200 years^1^. We are now living longer with a better quality of life than at any time in human history. However, disease-free lifespan has not increased as compared to the total lifespan, and an average of 16%–20% of our lives is spent with late-life morbidities^2,3^. The incidence of musculoskeletal disease, including spinal disorders, is increasing with the growing aging population. Adult spinal deformity (ASD) has gained significant attention in the last 10 years following improvements in diagnostic tools, classification schemes, and surgical techniques^4^. Furthermore, sagittal malalignment is widely known to correlate with clinical outcomes such as low back pain, and health-related quality of life (HRQOL) in patients with lumbar degenerative disorders, and especially ASD^5,6^. Non-surgical treatment is effective in ASD patients if they do not have significant radicular or mechanical back pain and have scoliosis less than 30° or subluxation less than 2 mm^7^. However, previous studies have reported that non-surgical treatment has not been successful for low back pain and radicular pain caused by ASD^8^. Some studies have demonstrated that surgical treatment is superior to non-surgical treatment in improving ASD patient outcomes^9,10^. With advances in minimally invasive surgery, represented by extreme lateral interbody fusion^11^, elderly people or high-risk patients who were previously inoperable can now undergo surgery. This implies that surgery for ASD is also expected to extend the healthy life span of elderly people. Although spinal fusion improves related symptoms and HRQOL in ASD patients, the loss of flexibility of the spinal column makes it difficult to perform daily activities that require trunk flexion^12^. Therefore, not all patients who receive surgical treatment for ASD obtain satisfactory results. Identifying preoperative factors that can predict surgical outcomes and patient satisfaction is very important in treating ASD. Many clinicians have reported about predictors of complications as well as mortality following ASD surgery^13,14^. However, limited information exists on preoperative predictors of surgical outcomes or patient satisfaction. In 2012, Miyamoto et al.^15^ found a skin abnormality, known as a Kitchen Elbow Sign (KE-Sign) (Fig. 1) on the extensor surface of the elbows and forearms of patients with intractable low back pain in the standing position. They reported that the KE-Sign is a skin change that runs from the elbow to the forearm in patients with intractable low back pain in the standing position, and is caused by supporting oneself using the elbows in a standing position during housework (e.g., during kitchen work). The KE-Sign is not specific to ASD patients, but may act as a surrogate marker of maintaining and continuing an independent lifestyle even with intractable low back pain due to sagittal plane imbalance if the population is limited to patients with ASD. Therefore, we hypothesized that the KE-Sign correlates with HRQOL and can be used to predict surgical outcomes or patient satisfaction in ASD. This study aimed to investigate the significance of KE-Sign in surgical cases of ASD.

## Materials and methods

We hypothesized that KE-Sign can be used to predict surgical outcomes and patient satisfaction in ASD.

This study was reviewed and approved by the local ethics committee (Research Ethics Committee of Wakayama Medical University: approval number 2943) and has been performed in accordance with the ethical standards as laid down in the 1964 Declaration of Helsinki and its later amendments. Informed consent was obtained from all participants.

**Figure 2:**
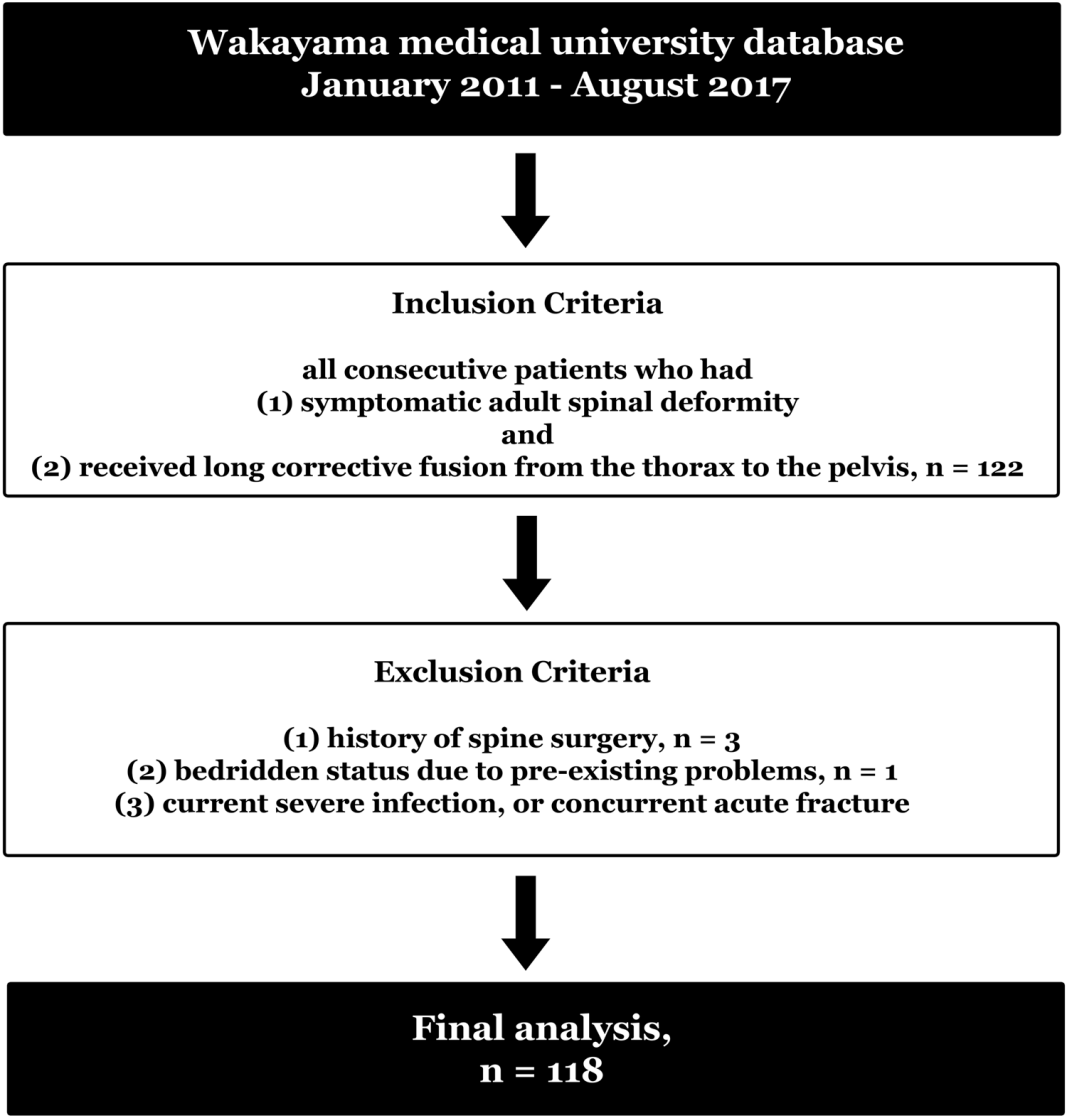
Flow diagram of the present study.

Between January 2015 and August 2019, all consecutive patients who had (1) symptomatic ASD and (2) received long corrective fusion from the thorax to the pelvis, were enrolled in this study. We defined ASD as C7 sagittal vertical axis (SVA) > 50 mm and pelvic incidence-lumbar lordosis (PI-LL) > 10° in accordance with the SRS-Schwab Classification^16^. The exclusion criteria were as follows: (1) history of spine surgery, (2) bedridden status due to pre-existing problems, (3) current severe infection, or concurrent acute fracture. The initial criteria for inclusion were met by 118 cases. Three patients who had a history of cervical spinal surgery were excluded, and after excluding 1 patient because of cerebral infarction, the remaining 114 patients were included in the final analysis (Fig. 2). The KE-Sign was evaluated at the outpatient clinic before surgery in all cases. Of the 3 parameters: (1) intractable low back pain in standing position, (2) pigmentation of dorsal forearm by visual inspection and (3) rough skin on the dorsal forearm by palpation, KE-Sign positive was defined as always satisfying (1) and satisfying any one of (2) and (3).

Prior to this study, 50 patients were examined by two orthopedic surgeons to determine inter-rater reliability of this definition by using Cohen’s Kappa test. The inter-rater reliability was Kappa 0.96.

### Clinical outcome measurements

Among indicators used to evaluate surgical outcomes of ASD, the Oswestry Disability Index (ODI) is the most commonly used^17^. The ODI was determined for every patient in our study^18^. It has 10 subclasses that measure pain intensity, personal care, lifting, walking, sitting, standing, sleeping, social life, sex life, and traveling. For each subclass, scores range from 0 (best measured health) to 5 (worst measured health). In this study, we used the Japanese version of ODI^19^ that excluded components related to sex life because limited information is expected for this component because of the Japanese cultural background; thus, the final score may be calculated as follows: (total score/(5 × 9 questions excluding “sex life”)) × 100%). ODI change score was calculated as preoperative ODI%–12-month postoperative ODI%. Moreover, the achievement rate of minimum clinically important difference (MCID) of ODI change score was evaluated with reference to the result of the previous study^20^. In order to evaluate patient satisfaction, we used the satisfaction visual analog scale (VAS). A 100-mm VAS has word descriptors “very dissatisfied” to “very satisfied” on the left and right, respectively. Subsequently, all scales were measured from the left to right to obtain the numeric values as the scores for patients’ satisfaction^21^.

We evaluated age, sex, body mass index (BMI), and presence or absence of KE-Sign as baseline characteristics of the patients; C7 SVA as an indicator of the whole spine alignment; PI-LL as an indicator of lumbar pelvis sagittal plane alignment; and ODI, lumbar VAS (low back pain and leg pain), and SF-36 as patient-based items. The SF-36 Physical Component Summary (PCS) and Mental Component Summary (MCS) scores were obtained using reported algorithms from a previous questionnaire^22^. KE-Sign was evaluated only before surgery while satisfaction VAS was evaluated only after surgery. All other parameters were evaluated before and 1 year after surgery. All results were collated and analyzed independently of the operating surgeon.

### Statistical analysis

To compare data between the KE-Sign positive and KE-Sign negative groups, Fisher’s exact test/chi square test was used for proportional variables. Analysis of variance (ANOVA) was performed for continuous variables. First, baseline characteristics and postoperative outcomes at 1 year after surgery were compared between the two groups. Then, for intragroup comparisons between baseline and one-year post-surgery, a paired t-test was used. The intervention effect (the change of each parameter including interaction) was also compared by a multivariate analysis of variance (MANOVA). Finally, multi-regression analysis was performed to identify predictors of patient satisfaction and improvement in ODI as dependent variables, and sex, age, BMI, presence or absence of KE-Sign, preoperative C7 SVA, PI-LL, ODI (%), lumbar VAS, and component summary scales of SF-36 as independent variables. All statistical analyses were performed using JMP Pro version 14 (SAS Inc., Cary, NC, USA), with the level of significance set at *P* < 0.05.

## Results

### Participant selection and comparison of preoperative items between KE-Sign positive group and KE-Sign negative group

One hundred and eighteen cases met the initial criteria for inclusion. Three patients who had a history of cervical spinal surgery were excluded, and after excluding 1 patient because of cerebral infarction, 114 patients were included in the final analysis (Fig. 2). The average age of the 114 patients was 70.8 years (± 6.6) and the majority (106/114) were female. Their average BMI was 23.3 kg/m^2^ (± 2.7), and the majority (92.1%) had Th10-S2 fusion range. Moreover, 58 patients were KE-Sign positive; 3 were male and 55 were female (Table 1). Preoperative age, BMI, C7 SVA, PI-LL, lumbar VAS, SF-36 component summary scales, and ODI% were not significantly different between the KE-Sign positive and KE-Sign negative groups (Table 1).

**Table 1:** Preoperative characteristics of the participants.

Items	KES ( +)	KES (−)	*P*-value
Number of patients	58	56	
Sex (male/female)	3/55	5/51	0.433
Age (years)	70.5 ± 6.7	71.1 ± 6.6	0.607
BMI (kg/m^2^)	23.0 ± 2.7	23.5 ± 2.8	0.184
C7 SVA (mm)	118.5 ± 50.0	125.0 ± 48.9	0.337
PI-LL (°)	42.0 ± 20.2	46.3 ± 19.9	0.363
VAS (low back pain) (mm)	62.7 ± 22.8	57.9 ± 24.8	0.393
VAS (leg pain) (mm)	41.3 ± 30.5	49.6 ± 30.4	0.234
SF-36 (PCS)	30.1 ± 13.8	28.4 ± 12.9	0.598
SF-36 (MCS)	66.8 ± 8.6	64.2 ± 8.3	0.108
ODI (%)	47.0 ± 15.1	47.6 ± 18.4	0.628

BMI, body mass index; SVA, sagittal vertical axis; PI-LL, pelvic incidence-lumbar lordosis; VAS, visual analog scale; SF-36, MOS 36-Item Short-Form Health Survey; PCS, physical component summary scale; MCS, mental component summary scale; ODI, Oswestry Disability Index.

**Significance defined as *P* < 0.05, *Significant trend as 0.05 < p ≤ 0.2, Data are presented as n, mean ± standard deviation (range), or number.

### Comparison of the one-year postoperative outcomes between KE-Sign positive group and KE-Sign negative group

All evaluated items including C7 SVA, PI-LL, VASs of low back pain and leg pain, SF-36 component summary scales, and ODI score were significantly improved from the baseline in both groups. Postoperative C7 SVA, PI-LL, VASs of low back pain and leg pain, and SF-36 component summary scales were not significantly different between the two groups (Table 2). However, the ODI score was significantly different between the two groups at 1 year after surgery. Furthermore, the MCID achievement rate of ODI score was significantly higher in the KE-Sign positive group (*P* = 0.003) (Table 2). Patient satisfaction VAS was significantly higher in the KE-Sign positive group (*P* < 0.001) (Table 2). Based on the above, the KE-Sign positive group showed better surgical outcomes and patient satisfaction than the KE-Sign negative group.

**Table 2:** Comparison of the 1-year postoperative outcomes between the KES positive group and KES negative group.

Evaluated items	KES ( +)	KES (−)	*P-*value
C7 SVA (mm)	38.2 ± 28.0	39.6 ± 32.7	0.779
PI-LL (°)	8.4 ± 9.4	9.4 ± 10.4	0.638
VAS (low back pain) (mm)	19.1 ± 15.1	19.5 ± 16.9	0.894
VAS (leg pain) (mm)	22.8 ± 17.0	21.4 ± 15.3	0.809
SF-36 (PCS)	32.7 ± 14.5	32.9 ± 15.7	0.962
SF-36 (MCS)	72.9 ± 14.1	67.8 ± 14.3	0.070
ODI (%)	18.8 ± 14.6	30.4 ± 18.5	0.001*
Improving of ODI (%)	28.2 ± 15.2	17.3 ± 18.1	0.001*
Achievement rate of MCID in ODI (%)	72.4 (42/58)	44.6 (25/56)	0.003*
Patient satisfaction VAS (mm)	92.3 ± 11.8	79.5 ± 19.4	< 0.001*

SVA, sagittal vertical axis; PI-LL, pelvic incidence-lumbar lordosis; VAS, visual analog scale; SF-36, MOS 36-Item Short-Form Health Survey; PCS, physical component summary scale; MCS, mental component summary scale; ODI, Oswestry disability index; Improving of ODI, preoperative ODI (%)–12-month postoperative ODI (%); MCID, minimum clinically important difference ODI change score (%) ≥ 12.8.

*Significant defined as *P* < 0.05, Data are presented as n, mean ± standard deviation (range).

### Intergroup comparison of the change of each parameter

Changes in each parameter were compared between the KE-Sign positive group and KE-Sign negative group (Table 3). The effect of time (i.e., surgical intervention) was significant for C7 SVA, PI-LL, lumbar VASs, SF-36 component summary scales, and ODI score. Moreover, ODI scores showed significant differences in group, and interaction.

**Table 3:** Intergroup comparison of the change in each parameter by MANOVA.

	Group *P*-value	Time (1 year) *P*-value	Interaction *P*-value
C7 SVA (mm)	0.509	< 0.001*	0.605
PI-LL (°)	0.254	< 0.001*	0.376
VAS (low back pain) (mm)	0.452	< 0.001*	0.280
VAS (leg pain) (mm)	0.347	< 0.001*	0.073
SF-36 (PCS)	0.769	< 0.001*	0.345
SF-36 (MCS)	0.047*	< 0.001*	0.232
ODI (%)	0.028*	< 0.001*	< 0.001*

MANOVA, Multivariate analysis of variance; SVA, sagittal vertical axis; PI-LL, pelvic incidence-lumbar lordosis; VAS, visual analog scale; SF-36, MOS 36-Item Short-Form Health Survey; PCS, physical component summary scale; MCS, mental component summary scale; ODI, Oswestry Disability Index.

*Significant defined as *P* < 0.05, Data are presented as n, mean ± standard deviation (range).

### Predictive factors for improving of ODI

Multiple regression analysis showed that KE-Sign positivity and preoperative ODI score were the significant factors predicting the ODI improvement. The standard partial regression coefficients were 0.30 and 0.61, respectively (Table 4).

**Table 4:** Multiple regression analysis for improvement in ODI.

Preoperative factor	*P*-value	Std. β	VIF
Sex	0.374	0.068	1.066
Age (years)	0.064	− 0.156	1.263
BMI (kg/m^2^)	0.664	− 0.034	1.088
KES ( +)	< 0.001*	0.300	1.121
SVA (mm)	0.411	− 0.076	1.572
PI-LL (°)	0.382	− 0.082	1.604
ODI (%)	< 0.001*	0.608	1.339
VAS (low back pain) (mm)	0.099	− 0.147	1.426
VAS (leg pain) (mm)	0.862	− 0.016	1.552
SF-36 (PCS)	0.154	0.111	1.101
SF-36 (MCS)	0.343	0.074	1.116

Std. β, Standard partial regression coefficient; VIF, variance inflation factor; KES, Kitchen Elbow Sign; BMI, body mass index; SVA, sagittal vertical axis; PI-LL, pelvic incidence-lumbar lordosis; VAS, visual analog scale; SF-36, MOS 36-Item Short-Form Health Survey; PCS, physical component summary scale; MCS, mental component summary scale; ODI, Oswestry Disability Index.

*Significant defined as *P* < 0.05.

### Predictive factors for postoperative patient satisfaction

Again, multiple regression analysis showed that age, KE-Sign positivity, and preoperative VASs of low back pain and leg pain were the significant factors in predicting the patient’s satisfaction at 1-year post- surgery. The standard partial regression coefficients were 0.29, 0.43, − 0.23, and 0.22, respectively (Table 5). Thus, older age, high leg pain intensity, and presence of KE-Sign predicted high satisfaction while high low back pain intensity predicted low satisfaction.

**Table 5:** Multiple regression analysis for patient satisfaction VAS.

Preoperative factor	*P*-value	Std. β	VIF
Sex	0.981	0.002	1.066
Age (years)	0.003*	0.287	1.263
BMI (kg/m^2^)	0.332	− 0.089	1.088
KES ( +)	< 0.001*	0.426	1.121
SVA (mm)	0.431	0.083	1.572
PI-LL (°)	0.446	− 0.081	1.604
ODI (%)	0.501	− 0.065	1.339
VAS (low back pain) (mm)	0.025*	− 0.228	1.426
VAS (leg pain) (mm)	0.039*	0.218	1.552
SF-36 (PCS)	0.301	0.091	1.101
SF-36 (MCS)	0.766	− 0.028	1.116

Std. β, Standard partial regression coefficient; VIF, variance inflation factor; KES, Kitchen Elbow Sign; BMI, body mass index; SVA, sagittal vertical axis; PI-LL, pelvic incidence-lumbar lordosis; VAS, visual analog scale; SF-36, MOS 36-Item Short-Form Health Survey; PCS, physical component summary scale; MCS, mental component summary scale; ODI, Oswestry Disability Index.

*Significant defined as *P* < 0.05.

## Discussion

The KE-Sign is often observed in patients who live an independent life. Until we obtained the results of this study, we believed that the KE-Sign correlates with HRQOL. Therefore, we hypothesized that the KE-Sign can be used to predict surgical outcomes or patient satisfaction in ASD. However, there are no reports investigating the relationship between the KE-Sign and ASD surgical outcomes and patient satisfaction. Therefore, we compared preoperative and postoperative parameters between the two groups, and performed multi-regression analysis for patient satisfaction and improvement in ODI, in order to investigate the significance of the KE-Sign in surgical cases of ASD. In this study, none of the preoperative characteristics of the patients were significantly different between the two groups. On the other hand, at the time of evaluation 1 year after surgery, the KE-Sign positive group showed significantly better surgical outcomes and patient satisfaction than the KE-Sign negative group. Moreover, in multi-regression analysis, KE-Sign positivity was significantly associated with both improvement in ODI and high satisfaction. These results suggest that the presence of the KE-Sign may be a predictor of high satisfaction and good surgical outcome. In other words, if KE-Sign exists preoperatively, good clinical results may be expected. We do not believe that the KE-Sign is specific to ASD patients or superior to other predictive parameters; nevertheless, we consider it to be clinically useful as a surrogate marker to further increase postoperative satisfaction if the target is limited to patients with ASD. Moreover, the KE-Sign can be easily evaluated visually. We speculate that the mechanism of KE-Sign development in ASD patients is as follows. Patients with ASD cannot perform sufficient outdoor activities without some form of support, such as carts or canes. Crucially, when patients use a cane or cart, they hold them in their hands so that their elbows are not pressed. On the other hand, when doing household chores while standing, such as kitchen work or ironing, ASD patients need to lean their elbows on a counter or table. The need to do household chores depends on each patient. In other words, it is assumed that preoperative KE-Sign ( +) reflects restrictions on both indoor and outdoor activities of daily living (ADL) while KE-Sign (−) reflects restrictions on outdoor ADL only. It is reasonable to believe that patients with both indoor and outdoor ADL restrictions that are improved after surgery would be more satisfied than patients that only experience improvement in outdoor ADL restrictions. Accordingly, we believe that KE-Sign ( +) can be a surrogate marker of good surgical indication for ASD and patient satisfaction. There was no significant difference between the KE-Sign ( +) and KE-Sign (−) groups in preoperative evaluation results, including pain intensity, SF-36 component summary scales, and ODI score. However, these measures do not sufficiently evaluate the ability to perform household chores. Therefore, we believe that KE-Sign may act as a surrogate marker of increased postoperative satisfaction with respect to the preoperative inability to perform household chores.

Previous studies, which examined preoperative predictors of surgical outcomes after spinal long fusion surgery, have not shown a significant correlation between age, BMI, spine fusion range, operative time, blood volume, and preoperative PI-LL mismatch^13,14^. In some reports, patients with high preoperative ODI scores had significant improvement in ODI after surgery^23^. However, other reports have shown that patients with high preoperative ODI score have poor surgical outcome^24^. In addition, since patient satisfaction is affected by the patient’s mental and physical health, the physician–patient relationship, incidence of perioperative complications, quality of the perioperative nursing care, and the patient’s expectations, among others, it is difficult to determine the predictors of patient satisfaction^25^. Only limited information exists on preoperative predictors of surgical outcomes or patient satisfaction. This study is the first report investigating the relationship between KE-Sign and surgical outcomes of ASD and patient satisfaction in surgical cases of ASD. This study suggests that KE-Sign is a preoperative factor that could predict both a high postoperative satisfaction level and good surgical outcomes.

Nevertheless, this study had some limitations. First, this study follows a retrospective design; thus, further studies will be needed to compare this patient group as the size of this patient population increases. Second, this study only addressed the effect of preoperative patient and surgical characteristics on 12-month postoperative patient satisfaction and surgical outcome, but did not address the effect of postoperative factors on long-term satisfaction and outcomes. Third, a globally standardized diagnostic criterion of KE-Sign is not established. Since KE-Sign is a new concept, there are still many unclear points about the pathology and significance of KE-Sign with ASD. There is a possibility that the results of this study cannot be generalized to other countries because the target population was limited to Japanese patients. However, since the Japanese lifestyle has already been undergoing westernization for decades, we believe that the results of this study will be reproduced in other countries as well. However, further research is needed to determine whether the results of this study can be adapted to other countries in which lifestyle and social welfare system are different from Japan. Fourth, the possibility that multiple factors may have confounded the relationship between the preoperative KE-Sign and postoperative results cannot be completely ruled out. We also did not report radiographic parameters, such as T1PA, GSA, C2-7SVA, C2-7 angle, T1-T12 angle, T11-L2 angle, and hip and knee angles, in this study because we intended to focus on the relationship between the KE-Sign and surgical outcomes, mainly in terms of HRQOL. The results of this study indicated that the KE-Sign ( +) predicted better improvement of ODI scores in ASD patients. However, HRQOL measures other than the ODI, such as the SRS-22, were not routinely evaluated during the study period. Future studies will need to evaluate the relationship between the KE-Sign and the radiographic parameters and quality of life scores that were not investigated in this study.

## Conclusions

Both groups with and without KE-Sign showed similarly good recoveries of SVA, PI-LL, lumbar VAS, and component summary scales of SF-36 postoperatively. However, improvement in ODI (%) and the VAS for satisfaction were significantly superior in KE-Sign positive patients. Thus, the KE-Sign may be useful as a surrogate marker of increased postoperative satisfaction in ASD patients with respect to the preoperative inability to perform household chores.

## References

[1] Oeppen J, Vaupel JW (2002). Demography. Broken limits to life expectancy. Science.

[2] Crimmins EM (2015). Lifespan and healthspan: past, present, and promise. Gerontologist.

[3] Jagger C, Gillies C, Moscone F, Cambois E, Van Oyen H, Nusselder W, Robine JM, EHLEIS team (2008). Inequalities in healthy life years in the 25 countries of the European Union in 2005: a cross-national meta-regression analysis. Lancet.

[4] Smith JS, Shaffrey CI, Ames CP, Lenke LG (2019). Treatment of adult thoracolumbar spinal deformity: past, present, and future. J. Neurosurg. Spine.

[5] Ames CP, Smith JS, Scheer JK, Bess S, Bederman SS, Deviren V, Lafage V, Schwab F, Shaffrey CI (2012). Impact of spinopelvic alignment on decision making in deformity surgery in adults: a review. J. Neurosurg. Spine.

[6] Endo K, Suzuki H, Tanaka H, Kang Y, Yamamoto K (2010). Sagittal spinal alignment in patients with lumbar disc herniation. Eur. Spine J..

[7] Everett CR, Patel RK (2007). A systematic literature review of nonsurgical treatment in adult scoliosis. Spine (Phila Pa 1976).

[8] Bridwell KH, Baldus C, Berven S, Edwards C, Glassman S, Hamill C, Horton W, Lenke LG, Ondra S, Schwab F, Shaffrey C, Wootten D (2010). Changes in radiographic and clinical outcomes with primary treatment adult spinal deformity surgeries from two years to three- to five-years follow-up. Spine (Phila Pa 1976).

[9] Kelly MP, Lurie JD, Yanik EL, Shaffrey CI, Baldus CR, Boachie-Adjei O, Buchowski JM, Carreon LY, Crawford CH, Edwards C, Errico TJ, Glassman SD, Gupta MC, Lenke LG, Lewis SJ, Kim HJ, Koski T, Parent S, Schwab FJ, Smith JS, Zebala LP, Bridwell KH (2019). Operative versus nonoperative treatment for adult symptomatic lumbar scoliosis. J. Bone Joint Surg. Am..

[10] Smith JS, Lafage V, Shaffrey CI, Schwab F, Lafage R, Hostin R, O’Brien M, Boachie-Adjei O, Akbarnia BA, Mundis GM, Errico T, Kim HJ, Protopsaltis TS, Hamilton DK, Scheer JK, Sciubba D, Ailon T, Fu KM, Kelly MP, Zebala L, Line B, Klineberg E, Gupta M, Deviren V, Hart R, Burton D, Bess S, Ames CP, International Spine Study Group (2016). Outcomes of operative and nonoperative treatment for adult spinal deformity: a prospective, multicenter, propensity-matched cohort assessment with minimum 2-year follow-up. Neurosurgery.

[11] Ozgur BM, Aryan HE, Pimenta L, Taylor WR (2006). Extreme lateral interbody fusion (XLIF): a novel surgical technique for anterior lumbar interbody fusion. Spine J..

[12] Hart RA, Gundle KR, Pro SL, Marshall LM (2013). Lumbar stiffness disability index: pilot testing of consistency, reliability, and validity. Spine J..

[13] Acosta FL, McClendon J, O'Shaughnessy BA, Koller H, Neal CJ, Meier O, Ames CP, Koski TR, Ondra SL (2011). Morbidity and mortality after spinal deformity surgery in patients 75 years and older: complications and predictive factors. J. Neurosurg. Spine.

[14] Buchlak QD, Yanamadala V, Leveque JC, Sethi R (2016). Complication avoidance with pre-operative screening: insights from the Seattle spine team. Curr. Rev. Musculoskelet. Med..

[15] Miyamoto K (2013). Elbows in elderly women with back pain; significance of skin examination on forearm association between Kitchen elbow sign (KE-Sign) and sagittal plane imbalance. Cent. Jpn. J. Orthop. Traumat..

[16] Schwab F, Patel A, Ungar B, Farcy JP, Lafage V (2010). Adult spinal deformity-postoperative standing imbalance: how much can you tolerate? An overview of key parameters in assessing alignment and planning corrective surgery. Spine (Phila Pa 1976).

[17] Cutler HS, Guzman JZ, Al Maaieh M, Connolly J, Skovrlj B, Cho SK (2015). Patient reported outcomes in adult spinal deformity surgery: a bibliometric analysis. Spine Deform..

[18] Fairbank JC, Pynsent PB (2000). The oswestry disability index. Spine (Phila Pa 1976).

[19] Fujiwara A, Kobayashi N, Saiki K, Kitagawa T, Tamai K, Saotome K (2003). Association of the Japanese Orthopaedic Association score with the Oswestry Disability Index, Roland-Morris Disability Questionnaire, and short-form 36. Spine (Phila Pa 1976).

[20] Copay AG, Glassman SD, Subach BR, Berven S, Schuler TC (2008). Minimum clinically important difference in lumbar spine surgery patients: a choice of methods using the Oswestry Disability Index, Medical Outcomes Study questionnaire Short Form 36, and Pain Scales. Spine J..

[21] Zhang XM, Shi JY, Gu YX, Qiao SC, Mo JJ, Lai HC (2017). Clinical investigation and patient satisfaction of short implants versus longer implants with osteotome sinus floor elevation in atrophic posterior maxillae: a pilot randomized trial. Clin. Implant. Dent. Relat. Res..

[22] Jenkinson C, Layte R, Lawrence K (1997). Development and testing of the Medical Outcomes Study 36-Item Short Form Health Survey Summary Scale Scores in the United Kingdom. Results from a large-Scale survey and a clinical trial. Med. Care.

[23] Than KD, Park P, Fu KM, Nguyen S, Wang MY, Chou D, Nunley PD, Anand N, Fessler RG, Shaffrey CI, Bess S, Akbarnia BA, Deviren V, Uribe JS, La Marca F, Kanter AS, Okonkwo DO, Mundis GM, Mummaneni PV, International Spine Study Group (2016). Clinical and radiographic parameters associated with best versus worst clinical outcomes in minimally invasive spinal deformity surgery. J. Neurosurg. Spine.

[24] Smith JS, Shaffrey CI, Lafage V, Schwab F, Scheer JK, Protopsaltis T, Klineberg E, Gupta M, Hostin R, Fu KM, Mundis GM, Kim HJ, Deviren V, Soroceanu A, Hart RA, Burton DC, Bess S, Ames CP, International Spine Study Group (2015). Comparison of best versus worst clinical outcomes for adult spinal deformity surgery: a retrospective review of a prospectively collected, multicenter database with 2-year follow-up. J. Neurosurg. Spine.

[25] Bess S, Akbarnia BA, Thompson GH, Sponseller PD, Shah SA, El Sebaie H, Boachie-Adjei O, Karlin LI, Canale S, Poe-Kochert C, Skaggs DL (2010). Complications of growing-rod treatment for early-onset scoliosis: analysis of one hundred and forty patients. J. Bone Joint Surg. Am..

